# Ozz-E3 Ubiquitin Ligase Targets Sarcomeric Embryonic Myosin Heavy Chain during Muscle Development

**DOI:** 10.1371/journal.pone.0009866

**Published:** 2010-03-24

**Authors:** Yvan Campos, Xiaohui Qiu, Edmar Zanoteli, Simon Moshiach, Naja Vergani, Antonella Bongiovanni, A. John Harris, Alessandra d'Azzo

**Affiliations:** 1 Department of Genetics and Tumor Cell Biology, St. Jude Children's Research Hospital, Memphis, Tennessee, United States of America; 2 Istituto di Biomedicina e di Immunologia Molecolare, Consiglio Nazionale delle Ricerche, Palermo, Italy; 3 Department of Physiology, University of Otago Medical School, Dunedin, New Zealand; University of Cincinnati, United States of America

## Abstract

Muscle contractile proteins are expressed as a series of developmental isoforms that are in constant dynamic remodeling during embryogenesis, but how obsolete molecules are recognized and removed is not known. Ozz is a developmentally regulated protein that functions as the adaptor component of a RING-type ubiquitin ligase complex specific to striated muscle. *Ozz^−/−^* mutants exhibit defects in myofibrillogenesis and myofiber differentiation. Here we show that Ozz targets the rod portion of embryonic myosin heavy chain and preferentially recognizes the sarcomeric rather than the soluble pool of myosin. We present evidence that Ozz binding to the embryonic myosin isoform within sarcomeric thick filaments marks it for ubiquitination and proteolytic degradation, allowing its replacement with neonatal or adult isoforms. This unique function positions Ozz within a system that facilitates sarcomeric myosin remodeling during muscle maturation and regeneration. Our findings identify Ozz-E3 as the ubiquitin ligase complex that interacts with and regulates myosin within its fully assembled cytoskeletal structure.

## Introduction

Striated muscle cells exhibit the paradoxical association of a rigidly ordered fine structure with the ability to adapt their size and contractile properties during growth and development, or in response to changes in their patterns of use. Many sarcomeric proteins are developmentally expressed as a series of isoforms leading at maturation to patterns appropriate for slow or fast contraction, and aerobic or anaerobic metabolism. Accordingly, mechanisms must exist to enable replacement of isoforms while maintaining an almost crystalline regularity of structural pattern. The classic suggestion of how such mechanisms may operate is based on *in vitro* experiments where myosin monomers spontaneously polymerize to reach a dynamic equilibrium between fully polymerized myosin and a small pool of soluble monomers [1]. However, in a theoretical study, Davis concluded that a model based on kinetic parameters could not account for the rapid replacement of one myosin isoform by another that is seen *in vivo* [2].

The ubiquitin-proteasome system [3]–[5] is the prime candidate for targeted degradation of most soluble and myofibrillar proteins. In skeletal muscles, ubiquitination of muscle proteins to target them for proteolysis is an important component of cachexia and muscle atrophy [6], [7]. Evidence for ubiquitin-mediated degradation of myosin is mostly indirect, but the E3 ubiquitin ligases MuRF1, which is induced during muscle atrophy, and MuRF3 mediate the ubiquitination of soluble myosin *in vitro* [8], [9], binding to multiple sites near the head region of MyHC molecules. Ubiquitination by MuRF1 has recently been shown to regulate the disassembly and degradation of the myofibrillar proteins MyBP-C, MLC1, and MLC2; however, MyHC is not ubiquitinated by MuRF1 *in vitro* when associated in the actomyosin complex or in the intact myofibrils [10]. Interestingly, ubiquitin-dependent degradation has also been indirectly implicated in the regulation of myosin folding and assembly [11].

Ozz, also known as Neurl2 (Neuralized-like protein 2), is the substrate-binding component of a RING (Really Interesting New Gene)-type ubiquitin ligase complex, which comprises Elongin B/C (Elo B/C), Rbx1 and Cullin 5 (Cul5) [12]. The protein primary structure contains two Neuralized Homologous Repeats (NHR1 and NHR2) that serve as protein-protein interaction domains and a SOCS (Suppressor of Cytokine Signaling) box at the C-terminus for recognition by the Elo B/C subcomplex. Ozz expression is muscle-specific and upregulated during muscle fiber differentiation, but we show here that it is downregulated in muscle atrophy. To form an active E3 ligase, Ozz must assemble with the other components of the complex, a process that adds an extra tier to regulation of substrate recognition and ubiquitination by this ligase [12]. This is in contrast to the MuRF family of ubiquitin ligases, which are monomeric and can initiate ubiquitination immediately upon binding their substrates [8], [9], [13].

We have established that sarcolemmal-associated β-catenin is a substrate for Ozz-E3 and that *ozz^−/−^* mice develop overt sarcomeric defects, which we have attributed in part to the impaired turnover of β-catenin at the membrane of differentiating myofibers [12].

We report here that the sarcomeric embryonic myosin heavy chain (MyHC_emb_/Myh3) is a novel substrate of Ozz, which specifically recognizes the rod domain or tail region of this protein. MyHC_emb_ expression is associated with initiation of sarcomere formation [14], leading to the idea that it is optimized for self-assembly into new thick filaments followed by a sequence of subunit changes to give rise to adult myofilaments [15]. Embryonic muscles form in two stages: a small number of primary myotubes form a scaffold to direct the later formation of secondary myotubes, which give rise to the majority of adult muscle fibers [16]–[18]. MyHC_emb_, together with MyHC_slow_, is expressed during primary myotube formation, and again together with MyHC_neo_, during secondary myotube formation [19], [20]. It also is the first myosin isoform to be expressed when new myotubes form in regenerating adult muscle [21]; in intact adult muscles during hypertrophy induced by passive stretch [22]; or during recovery from immobilization-induced atrophy [23]. Shortly after birth it is rapidly replaced by adult myosin isoforms [20], [24].

Here we present evidence that the Ozz-E3 ligase, by binding to the rod domain of a fully assembled MyHC_emb_, marks it for ubiquitination and degradation, probably facilitating the subsequent assembly of new isoforms. These observations lead to the idea that in muscle tissue the ubiquitin-proteasome system, in addition to its involvement in atrophy, removal of misfolded/damaged proteins, and proper folding and assembly of structural proteins, may also facilitate exchange of isoforms within large polymeric assemblies to regulate tissue development, remodeling and regeneration.

## Results

### Ozz is Downregulated in Muscle Atrophy

**Figure 1:**
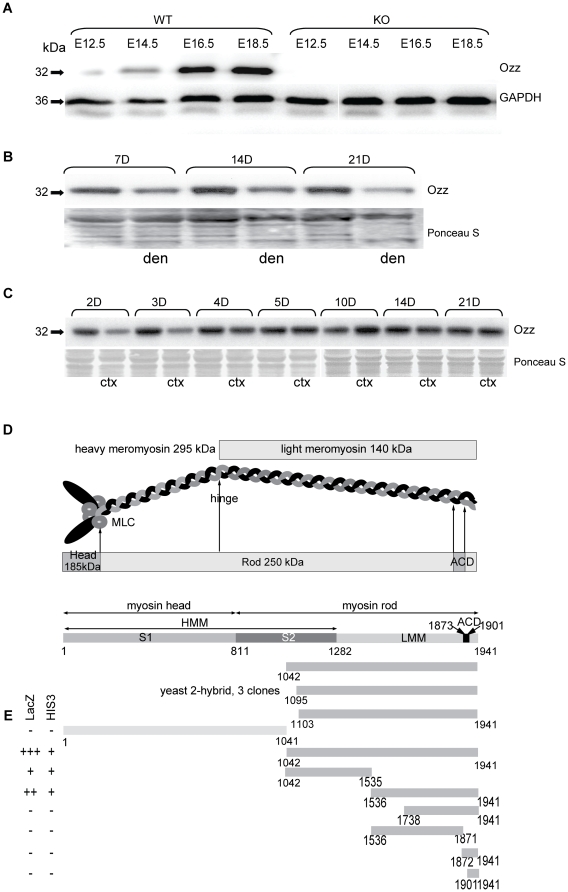
Ozz Expression Is Upregulated During Development and Regeneration, but not Atrophy. (A) Ozz is present during the initial phase of myogenesis (E12.5) and expression progressively increases during embryonic muscle development. (B) Expression of Ozz in the *gastrocnemius* muscle progressively decreases during denervation-induced muscle atrophy. (C) Ozz expression following cardiotoxin injection falls during the degeneration phase (2–4d post-injection) and then increases during the regeneration phase (5–10d post-injection) before returning to control levels. (D) Schematic diagram of a myosin molecule, depicting the position of the 3 cDNA clones within the tail portion of MyHC_emb_ isolated by a yeast 2-hybrid screen. (E) To map the Ozz binding region(s) on MyHC_emb_, various MyHC_emb_ constructs were screened against full length Ozz in a yeast 2-hybrid system. Two reporter genes (HIS3 and LacZ) were employed to assess protein-protein interaction. Amino acid residues are indicated. HMM, heavy meromyosin; LMM, light meromyosin; ACD, adhesion competence domain.

Up-regulation of ubiquitin ligases and protein ubiquitination are common correlates of muscle atrophy. We have shown earlier that the expression of *ozz* mRNA and Ozz protein increases during muscle development from embryonic day E12.5 onward [12] (Fig. 1A). We now wished to test if there was a similar response with muscle atrophy. Ozz levels progressively fell following denervation (Fig. 1B), suggesting the involvement of Ozz in muscle growth rather than atrophy. The latter conclusion was further supported by the pattern of Ozz expression upon injury of adult muscles with local injection of cardiotoxin. This procedure provokes initial profound muscle degeneration, followed by regeneration [21], [25]. Ozz expression was downregulated during the phase of degeneration, but was upregulated during the regeneration phase (Fig. 1C), confirming that Ozz expression is associated with muscle development and growth.

### Ozz Targets Embryonic Myosin Heavy Chain

In a yeast 2-hybrid screen of an E14.5 mouse cDNA library we identified MyHC_emb_ as a novel interaction partner of Ozz. Either full-length Ozz or the N-terminal half of the protein (residues 1–229), including the entire NHR1 domain (residues 14–104) and most of the NHR2 domain (residues 208–242) were used as baits. Both screens yielded 3 clones with 93% homology to MyHC_emb_ spanning residues 1042–1941 of the tail domain (Fig. 1D). To confirm Ozz' interaction with the tail of MyHC_emb_ and to identify the minimal regions of the tail needed for this interaction, we performed a series of 2-hybrid experiments using as preys either the full length tail domain (residues 1040–1941), the full length head/neck domain (residues 1–1040), or several deletion mutants of the tail region (Fig. 1E and Fig. S1A). These mutant peptides either included or excluded a 29 amino acids (1873–1901 aa) assembly competence domain (ACD) near the C-terminus of the myosin tail, which is responsible for proper myosin assembly into thick filaments [26]. We found that Ozz interacted strongly with the full-length tail, but not with the head/neck domain. Two deletion fragments of the myosin tail encompassing either the N-terminal amino acids 1041–1535, or the C-terminal amino acids 1536 to 1941 interacted differently with the full length Ozz: the former bound weakly, while the latter maintained a strong interaction (Fig. 1E and Fig. S1A). By further deleting the latter fragment at either its C-terminus (1738–1941 aa) or N-terminus (1536–1871 aa) we completely abolished Ozz binding. Similarly, two truncated fragments spanning amino acids 1872–1941, encompassing the ACD domain, also showed no interaction with Ozz. These results identified at least two regions of the MyHC_emb_ tail crucial for Ozz binding, which likely depends on the 3D folding of the MyHC_emb_ tail.

**Figure 2:**
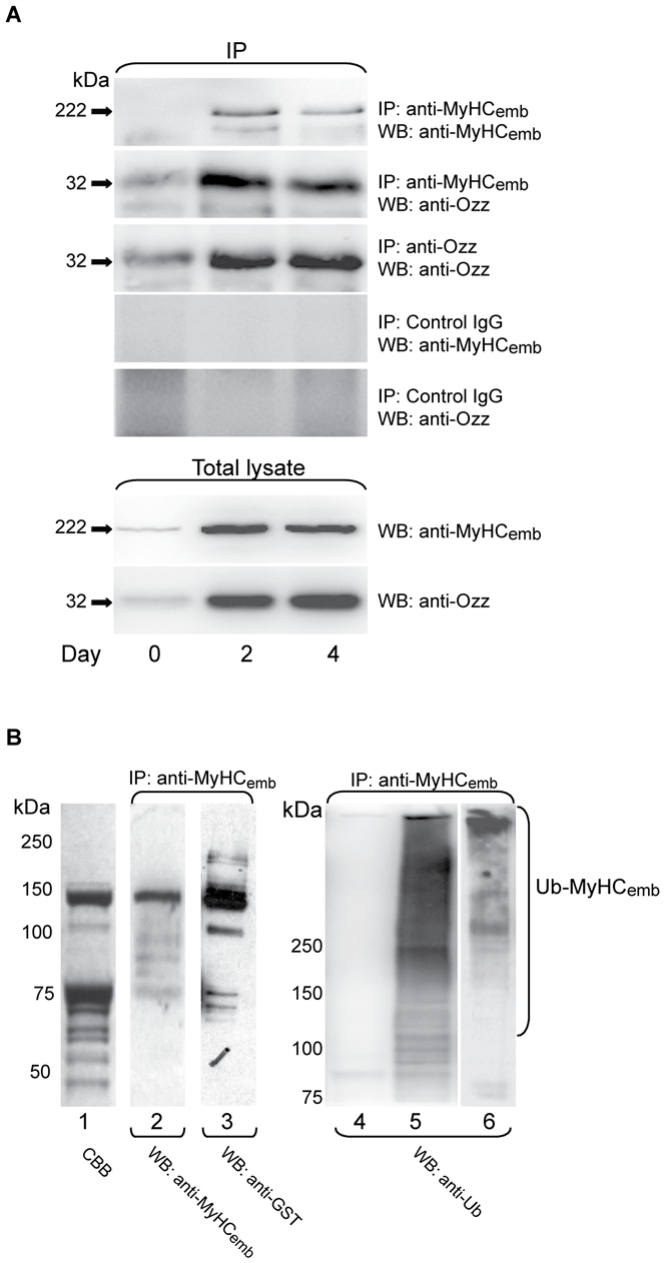
Ozz Binds MyHC_emb_ to Promote its Ubiquitination. (A) Lysates of undifferentiated cultured myoblasts (day 0) and differentiating or differentiated myotubes (days 2–4) were subjected to immunoprecipitation using anti-MyHC_emb_, anti-Ozz antibodies, or isotype matching IgG as control. Immunoblotting of the co-precipitated proteins as well as the total lysates with anti-MyHC_emb_ and anti-Ozz antibodies demonstrated that Ozz and MyHC_emb_ are bound in a stable complex. (B) *In vitro* ubiquitination of recombinant MyHC_emb_ mediated by Ozz-E3 ligase. A GST-tagged MyHC_emb_ fragment of ≈130 kDa (1041–1942 aa), which included the myosin rod region, was incubated with Ozz-E3 ligase and either native ubiquitin or a mutant Ub K48R. In the presence of Ozz-E3 ligase and native ubiquitin the MyHC_emb_ fragment was efficiently ubiquitinated. (Lane 1) Coomassie-stained gel of the GST-MyHC_emb_ fragment used as substrate for the reaction. (Lane 2) Immunoprecipitation of GST-MyHC_emb_ fragment with anti-MyHC_emb_ antibody, followed by immunoblotting with anti-MyHC_emb_. (Lane 3–6) Immunoprecipitation of the *in vitro* ubiquitinated GST-MyHC_emb_ fragment with anti-MyHC_emb_ antibody, followed by immunoblotting with anti-GST (3) or anti-Ubiquitin (4–6). *In vitro* ubiquitination reactions were as follows: (lane 4) Ozz-E3 + ubiquitin - MyHC_emb_; (lane 3 and 5) Ozz-E3 + ubiquitin + MyHC_emb_; (lane 6) Ozz-E3 + ubiquitin mutant K48R + MyHC_emb_.

To verify whether the Ozz-MyHC_emb_ interaction occurred *in vivo,* crude lysates of proliferating (day 0), differentiating (day 2) and terminally differentiated (day 4) primary myoblast cultures prepared from newborn wild-type mice were immunoprecipitated with anti-MyHC_emb_ antibody or an isotype matching control IgG, and probed on immunoblots with anti-Ozz antibody. The results showed that Ozz was effectively co-immunoprecipitated with MyHC_emb_, indicating that the two endogenous proteins were firmly linked (Fig. 2A).

We next tested if Ozz could exert its ubiquitin ligase activity towards MyHC_emb_. For this purpose we performed *in vitro* ubiquitination assays using a purified, reconstituted Ozz-E3 complex [12] and a GST-tagged MyHC_emb_ fragment spanning the tail portion of the protein (residues 1041–1942). The ubiquitinated products were then immunoprecipitated with anti-MyHC_emb_ and the immunoblots probed with anti-ubiquitin antibody or anti-GST antibody, used as control (Fig. 2B). Recombinant MyHC_emb_ tail and its proteolytic fragments of smaller molecular weight were ubiquitinated only in the presence of the Ozz-E3 complex (Fig. 2B, compare lanes 4 and 5). Furthermore, if the assay was performed using the ubiquitin mutant K48R to avoid the formation of a conjugated ubiquitin chain at this residue, ubiquitination was reduced to background levels (Fig. 2B, lane 6), demonstrating that Ozz-E3 polyubiquitinated the MyHC_emb_ tail. Five other GST fusion proteins, used as internal controls, were not ubiquitinated in this assay (data not shown), confirming the specificity of Ozz-E3 activity towards its substrate.

### Ozz Segregates with Fully Assembled Sarcomeric Myosin during Myofibrillogenesis

**Figure 3:**
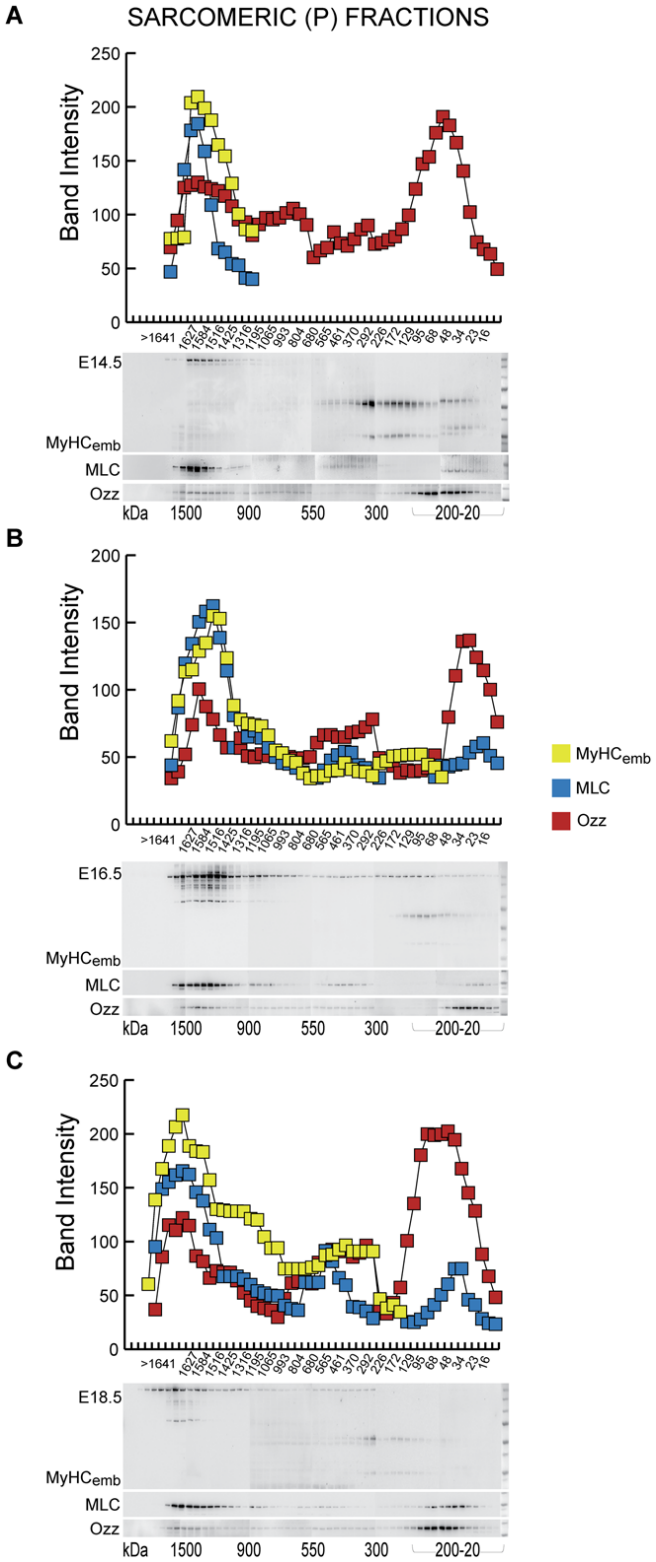
Ozz Segregates with Myofilamentous MyHC_emb_ from the Earliest Stages of Myofibrillogenesis. Sarcomeric (P) fractions from E14.5 (top), E-16.5 (middle), and E18.5 (bottom) embryonic muscles were separated according to their molecular size (horizontal axis) by gel filtration chromatography. Aliquots of each eluted fraction were separated by SDS-PAGE and then immunoblotted with anti-MyHC_emb_ (green), anti-Ozz (red), and anti-MLC (blue). The average intensities from three experiments were measured and plotted, as indicated. The densitometric analysis of the protein profile shows that the high molecular weight fractions (≈1500 kDa - ≈900 kDa) of sarcomeric (P) preparations contained polymeric myofilamentous MyHC_emb_, as confirmed by the presence of MLC. Ozz co-eluted with the high molecular weight myofilamentous MyHC_emb_ only in the sarcomeric (P) fractions, but was conspicuously absent from the cytoplasmic fractions (data not shown). Ozz and MLC also eluted in size fractions corresponding to their monomeric molecular weights, in both sarcomeric and cytoplasmic subcellular fractions (data not shown).

In a chaperone-mediated process [27]–[30] newly synthesized myosin isoforms are serially polymerized as monomers; dimers plus 4 myosin light chains (MLC) to form hexamers; and assembled thick filaments. To determine which pool of myosin is targeted by Ozz *in vivo* we first tested the chromatographic profiles of Ozz and MyHC_emb_ after gel filtration of muscle extracts from embryos of different stages (E14.5–E18.5). Myosin preparations conventionally employ high ionic strength extraction buffers to solubilize fibrillar myosin. Here we used differential centrifugation of muscle lysates of wild-type embryos in a buffer close to physiological ionic strength to obtain a supernatant containing mostly soluble myosin (S) and an insoluble high-speed pellet including sarcofilamentous myosin (P). Examination of the P myosin preparations with immunofluorescence microscopy confirmed the presence of fragments of sarcomeres staining positively for MyHC_emb_ and α-actinin (data not shown), which validated our extraction procedure. We then separated the P and S myosin preparations on gel filtration columns and assessed the levels and distribution of MyHC_emb_ and Ozz on immunoblots of the eluted fractions probed with anti MyHC_emb_, anti-Ozz and anti-MLC antibodies. The profiles shown in Fig. 3 (upper panels) were generated by densitometric analyses of band intensities.

Throughout myofibrillogenesis (E14.5-E18.5) full-length myosin eluted from the column mainly in two groups of fractions in the size ranges ≈1500–900 kDa and ≈800–500 kDa. These fractions from both sarcomeric (Fig. 3) and soluble myosin preparations (data not shown) contained MyHC_emb_ and MLC, indicating the presence of multimeric myosin (≈1500–900), myosin hexamers (2 heavy chains and 4 light chains, MW 520 kDa), as well as lower mw fragments. A third group of fractions, corresponding to sizes < ≈200 kDa, contained reproducible anti-MyHC_emb_ +ve bands of ≈50 kDa, 30 kDa and 25 kDa, evidently myosin peptides (Fig. 3). Their size distribution indicated that some passed through the column as dimers. The separation pattern of the different MyHC_emb_ bands varied only slightly among embryos of different ages.

In the same high molecular weight column fractions of sarcomeric preparations (P), a portion of Ozz consistently co-eluted with filamentous myosin, suggesting that Ozz is already bound to sarcomeric myosin during the early stages of myofibrillogenesis (E14.5, Fig. 3). In contrast, Ozz was totally absent from all fractions in the size range ≈1500–550 kDa from the S preparations (data not shown). Free Ozz eluted from the column in size fragments near its monomeric molecular weight of ≈31 kDa and was detected at comparable levels in both the P and S preparations.

**Figure 4:**
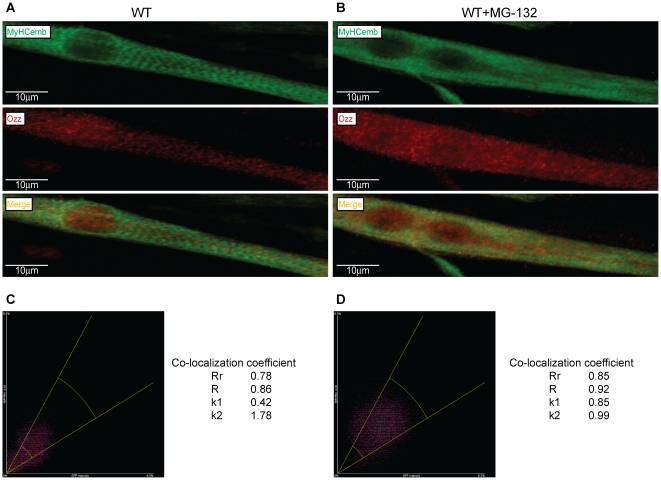
Co-localization of Ozz with Sarcomeric MyHC_emb_ during Myofiber Differentiation. (A) and (B) Confocal microscopy images of differentiating myotubes (day 4) untreated (A) and treated (B) with MG-132. Cells treated with the proteasome inhibitor showed a clear increase of co-localization of MyHC_emb_ and Ozz compared to untreated myotubes. (C) and (D) Computational analyses of confocal images of differentiated myotubes untreated (C) and treated (D) with MG132 confirmed the visualization of the co-localized fluorochromes. Pearson's correlation coefficient (Rr), Manders overlap (R), and Manders overlap coefficients *k1* and *k2* were employed to evaluate the extent of colocalization of the two fluorescent dyes.

The finding that Ozz co-elutes with the high molecular weight pool of MyHC_emb_ on size exclusion columns was further supported by immunofluorescence labeling of differentiated primary myotubes (day 4), treated and not treated with the MG-132 proteasome inhibitor, using anti-Ozz and anti-MyHC_emb_ antibodies (Fig. 4). Confocal microscopy and computational analyses of the two fluorescent signals indicated that a selected pool of sarcomeric MyHC_emb_ co-localized with Ozz in the untreated myotubes (Fig. 4A and C). Given that the co-localization of the two proteins increased substantially in fibers treated with the proteasome inhibitor, as determined by the co-localization coefficient (Fig. 4B and D), we can infer that Ozz regulates the proteasomal degradation of a selected pool of sarcomeric MyHC_emb_ during myofiber differentiation.

### Ozz Interacts and Ubiquitinates Fully Assembled Sarcomeric Myosin

**Figure 5:**
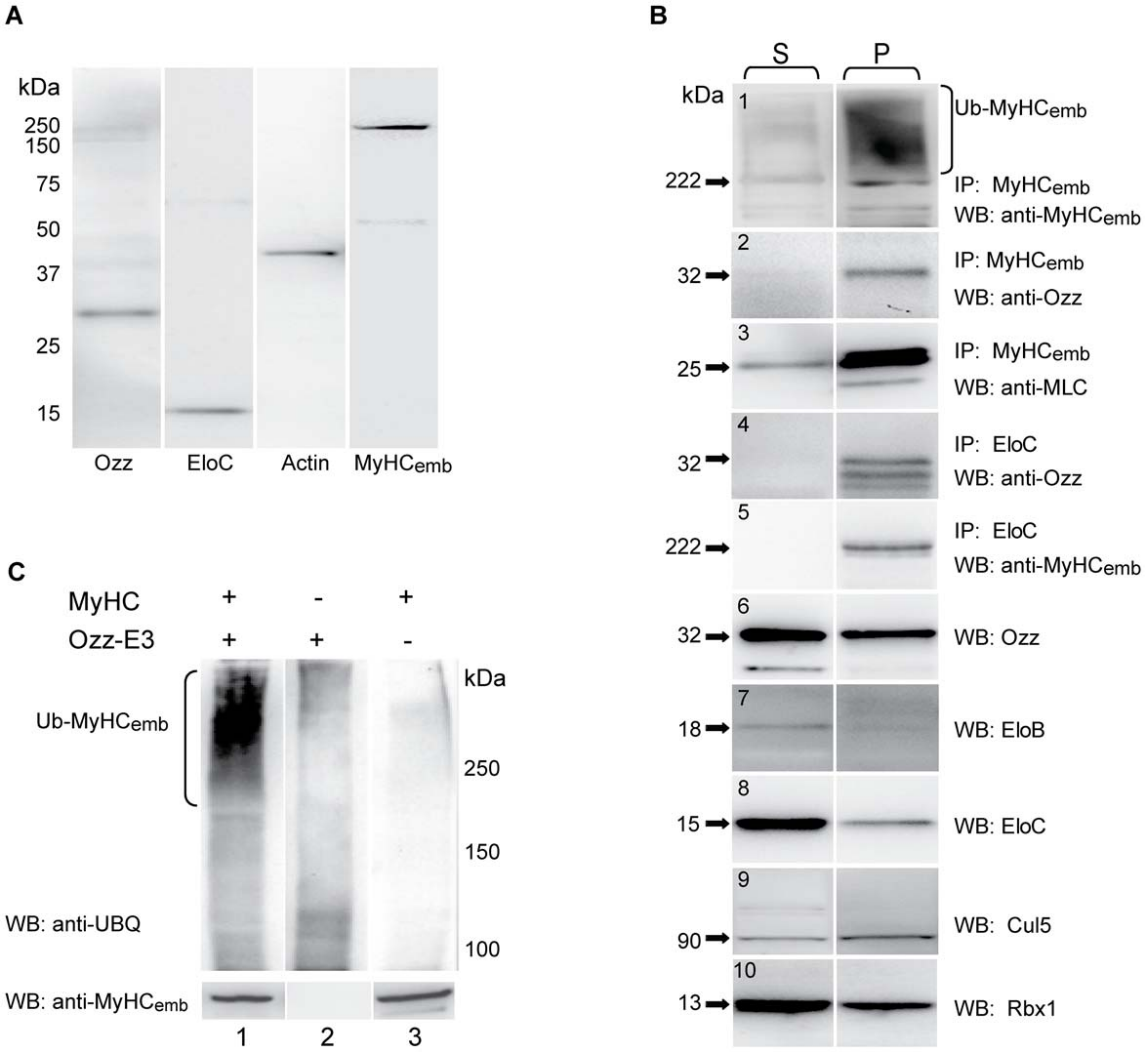
Ozz Interacts with Myofilamentous MyHC_emb_. (A) Ozz was recovered in a classical preparation of thin-thick filaments from E16.5 embryonic muscle, demonstrating that it is stably bound to sarcomeric myosin. Also, the presence of Elo C in this preparation indicates that the Ozz-Elo C sub-complex is associated with the myofilaments. (B) Cytoplasmic (S) and sarcomeric (P) fractions from wild-type E16.5 embryonic muscles were analyzed on Western blots for the presence of Ozz and its binding partners Elo B, Elo C, Cul5, Rbx1. The same preparations were also subjected to immunoprecipitation with anti-MyHC_emb_ or anti-Elo C, and the immunoprecipitates blotted and probed with anti-MyHC_emb_, anti-Ozz or anti-MLC. Ozz co-immunoprecipitated with MyHC_emb_ and Elo C only from the sarcomeric fractions, not the cytoplasmic fractions. (C) In vitro ubiquitination of native sarcofilamentous myosin purified from wild-type newborn muscle (P1). (Lane 1) Ozz-E3 efficiently ubiquitinated sarcomeric MyHC_emb_. The specificity of the reaction was confirmed by omitting either the substrate (lane 2) or the Ozz-E3 complex (lane 3) from the ubiquitination assay, which significantly reduced the Ub-MyHC_emb_ products. Note the similarity with (B) panel 1, identifying the high molecular weight smear as ubiquitinated MyHC_emb_.

Having established that a portion of the Ozz protein segregates with assembled MyHC_emb_, we wanted to ascertain whether Ozz was detectable in a classical preparation of muscle thin-thick filaments from E16.5 embryos [31], [32]. We chose E16.5 embryos because this embryonal stage coincides with the onset of secondary myogenesis. Western blot analysis of these preparations demonstrated the co-purification of Ozz and its direct interacting partner Elo C with the thin-thick filaments, indicating an association of the entire Ozz-E3 complex with fully assembled myosin (Fig. 5A). To further validate these results, we checked whether all components of the Ozz-E3 complex were bound to assembled myosin in the insoluble preparations (P) from muscles of E16.5 embryos. Direct interaction of Ozz with myofilamentous MyHC_emb_ was proven by co-immunoprecipitation of Ozz with anti-MyHC_emb_ antibody only from the insoluble preparations (P), but not from the soluble preparations (S), albeit the amount of Ozz was greater in the latter (Fig. 5B, panels 2 and 6). Together these data show that Ozz is bound in a stable form to assembled sarcomeric myosin since the early stages of myofibrillogenesis, but at an untraceable level to soluble myosin.

Fractions from both S and P preparations were also probed on immunoblots with antibodies against each component of the Ozz-E3 complex (Elo B/C, Cul5, and Rbx1). Notably, all 4 proteins were detected together with Ozz not only in the soluble pool (S) of extracted muscle proteins but also in the insoluble sarcomeric preparations from wild-type embryos (Fig. 5B, panels 7–10). Furthermore and in agreement with the observed presence of both Ozz and Elo C in purified thin-thick filaments (Fig. 5A), we found a portion of Ozz bound to Elo C within the myofibrils (P fraction) but not in the soluble (S) fraction (Fig. 5B, panel 4). To ascertain whether the other components of the Ozz-E3 complex associated with the Ozzylated sarcomeric MyHC_emb_, extracted muscle proteins were immunoprecipitated with anti-Elo C from the S and P fractions and probed on immunoblots with both anti-MyHC_emb_ and antibodies against the remaining E3 components. We found that MyHC_emb_ was effectively co-immunoprecipitated with anti-Elo C (Fig. 5B, panel 5). However, under these stringent experimental conditions (high salt concentration), while we were able to demonstrate Ozz binding with its direct interacting partners, MyHC_emb_ and Elo C, we could not co-immunoprecipitate the remaining components of the Ozz-E3 complex (not shown). Nonetheless, the presence in the sarcomeric, insoluble fractions of all Ozz partners and the demonstrated interaction of Ozz, Elo C and MyHC_emb_ strongly support the notion that the Ozz-E3 complex is assembled within the myofibrils.

Finally, to test whether MyHC_emb_ was efficiently ubiquitinated by Ozz-E3 when associated with the myofibril, we used sarcofilamentous myosin, purified from wild-type newborn muscle [33], [34], as substrate in an *in vitro* ubiquitination assay. We found that Ozz-E3 efficiently ubiquitinated the endogenous, assembled MyHC_emb_ (Fig. 5C). The specificity of the reaction was confirmed by the lack of ubiquitinated products in the absence of either the substrate (lane 2) or the Ozz-E3 complex (lane 3).

### MyHC_emb_ Expression Persists in *Ozz^−/−^* Muscle

**Figure 6:**
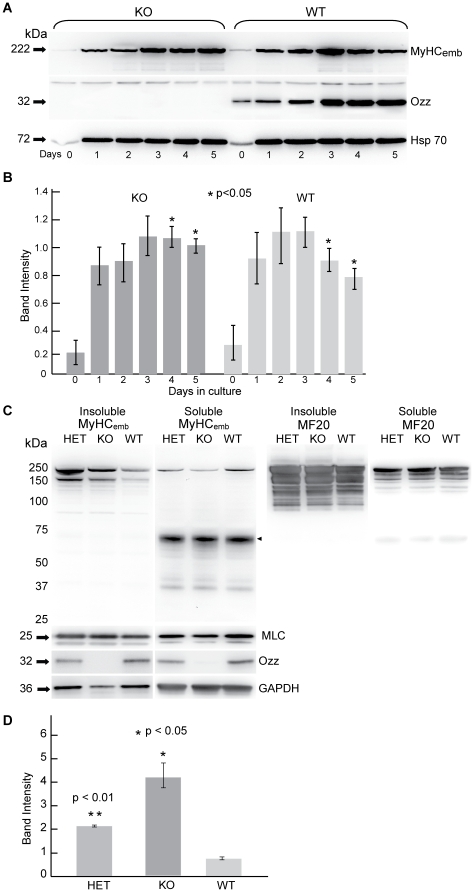
MyHC_emb_ Expression is Abnormally Prolonged in *Ozz*^−/−^*Mutants.* (A) MyHC_emb_ expression is prolonged during *in vitro* differentiation of *ozz^−/−^* primary myoblasts. Western blots showing the time course of MyHC_emb_ and Ozz expression in undifferentiated myoblasts (day 0) and differentiating or differentiated multinucleated myotubes (day 1–5) from null mutants and wild-type controls. (B) Quantification of MyHC_emb_ expression in the soluble fractions shown in (A), normalized against Hsp 70 levels. Data are expressed as mean ± SD of three independent experiments. Groups were compared by the Student t-test for two samples assuming equal variance. Mean differences were considered statistically significant when P values were less than 0.05 (*). (C) Western blot analyses of the sarcofilamentous, insoluble fractions of muscle isolated from *ozz^+/+^, ozz^+/–^,* and *ozz^−/−^* P7.5 pups showed that the expression of MyHC_emb_ is greater in the null and heterozygous samples than in the wild-type samples. In contrast, in the cytoplasmic, soluble fraction from the same preparations the expression of MyHC_emb_ is greater in the wild-type samples. Ozz expression is approximately halved in heterozygotes, while MLC expression remains normal. GAPDH is shown as loading control. (D) Quantification of MyHC_emb_ expression in the insoluble fractions shown in (C), standardized against GAPDH expression. Data are expressed as mean ± SD of three independent experiments. Groups were compared by the Student t-test for two samples assuming equal variance. Mean differences were considered statistically significant when P values were less than 0.05 (*).

Mouse myoblasts in primary culture multiply and then quickly fuse into multinucleated myotubes expressing MyHC_emb_ and other MyHC isoforms. Immunoblots of lysates of primary myoblasts induced to differentiate *in vitro* confirmed that Ozz expression was rapidly upregulated as myotubes formed (Fig. 6A). Normalization of immuno-positive myosin bands to the Hsp 70 loading control in three independent experiments showed that in wild-type myotubes MyHC_emb_ levels peaked at day 3 and then fell as expression of other isoforms increased (Fig. 6A and B); this pattern of expression has been described in both mouse and human muscle cultures [14], [35]. In *ozz^−/−^* myotubes, by contrast, at day 4 and day 5 of differentiation MyHC_emb_ levels remained significantly higher than in control myotubes (Fig. 6A and B). This observation was further confirmed by measuring the mean fluorescence intensity of individual wild-type and *ozz^−/−^* primary myotubes (day 4) (Fig. S2A and B). A significant increase in the fluorescence intensity of MyHC_emb_ was detected in *ozz^−/−^* myotubes compared to wild-type myotubes (Fig. S2B), suggesting improper regulation of this myosin isoform during myotube formation.

Shortly after birth, MyHC_emb_ expression normally falls rapidly, becoming undetectable within 2–3 weeks postnatal [20]. To test if expression of MyHC_emb_ protein was also disturbed in the *ozz^−/−^* mice, we compared the amount of MyHC_emb_ in *ozz^−/−^, ozz^+/–^* and *ozz^+/+^* muscles isolated from the limbs of 7.5-day-old mouse pups. Immunoblots and quantitative analyses of these preparations demonstrated that *ozz^−/−^* and heterozygous pups had a significantly higher proportion of MyHC_emb_ in the insoluble myosin fraction than their corresponding wild-type littermates (Fig. 6C and D). By contrast, wild-type soluble myosin had a relatively greater proportion of MyHC_emb_ than the corresponding *ozz* heterozygous and knockout samples (Fig. 6C, middle panel). A prominent proteolytic fragment of about 50 kDa was similar in all samples (Fig. 6C, middle panel, arrowhead). The levels of MLC in both insoluble and soluble fractions paralleled those of MyHC_emb_ (Fig. 6C, MLC). Total MyHC (antibody MF20) was used as additional control (Fig. 6C). Together these observations denote a phenotype of slowed release of MyHC_emb_ from sarcomeric myofilaments in postnatal *ozz^−/−^* muscle tissue. Heterozygotes, which have around half the normal level of expression of Ozz, also had increased levels of insoluble myosin, suggesting that Ozz may be regulatory as well as necessary for normal embryonic myosin isoform disassembly and replacement with the postnatal isoform. One possibility is that an Ozz molecule must attach to each monomer in a MyHC_emb_ dimer to efficiently promote its ubiquitination and removal, making the process sensitive to absolute levels of Ozz expression.

**Figure 7:**
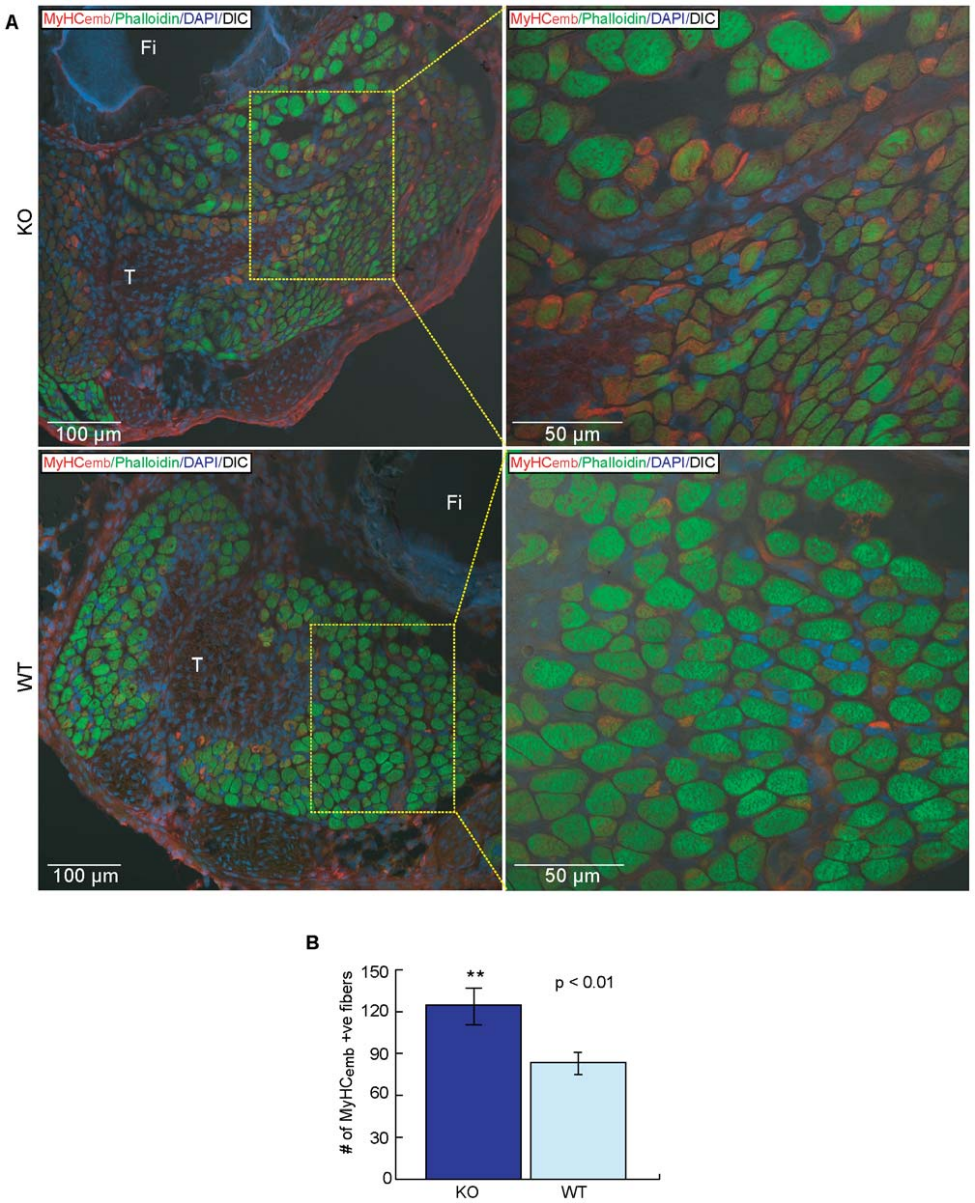
Prolonged Expression of MyHC_emb_ in Postnatal *Ozz^−/−^* Mice. (A) Expression of MyHC_emb_ in P7.5 null mutant and wild-type hind limbs. Representative cross sections of the *ozz* null and wild-type m. *gastrocnemius,* showing a portion of fibula (Fi) bone and including a portion of the intramuscular distal tendon (T), were stained with anti-MyHC_emb_ and phalloidin. The right panels represent higher magnification pictures of the areas boxed in yellow in the left panels. (B) Quantification of MyHC_emb_ positive fibers in P7.5 null mutant and wild-type muscles. Data are expressed as mean ± SD of three independent experiments. Groups were compared by the Student t-test for two samples assuming equal variance. Mean differences were considered statistically significant when P values were less than 0.05 (**).

These biochemical data were confirmed by immunofluorescence and confocal microscopy analyses of cross sections of the hind limbs from P7.5 wild-type and null pups. In *ozz^+/+^* muscles we found that MyHC_emb_ was in course of being removed and replaced by other MyHC isoforms. In fact, many fibers sectioned near the mid-belly region of individual wild-type muscles showed little or no expression of MyHC_emb_, which was instead more easily detected in sections near the muscle tendon region of the muscle, indicating a gradient in isoform displacement from the middle towards the ends of the muscle fibers. By contrast, matched sections of *ozz^−/−^* limbs displayed a higher number of MyHC_emb_ expressing fibers throughout the length of the muscles, but most prominently near the muscle tendon region. The sections were cut at the level of the distal third of the *gastrocnemius* and *tibialis anterior* muscles to include the intra-muscular tendons, and labeled with anti-MyHC_emb_ and phalloidin. Fig. 7A shows a high magnification representative of these sections at the level of the *gastrocnemius*. Contrary to the wild-type limb muscles (Fig. 7A, lower panels), every fiber close to the tendon in the *ozz^−/−^* muscles expressed high levels of MyHC_emb_ (Fig. 7A, upper panels) as did the majority of peripheral fibers of the same muscle. A quantification of the total number of MyHC_emb_ +ve fibers present throughout the length of the limbs is shown in Fig. 7B.

## Discussion

We have previously described the identification of Ozz as the substrate-recognition component of a striated muscle-specific RING-type E3 ubiquitin ligase complex, involved in myofiber differentiation [12]. Here, we present evidence that Ozz plays a critical role in muscle development and regeneration, but not in muscle atrophy. In addition, we found that during muscle differentiation Ozz recognizes the developmental isoform MyHC_emb_ as one of its substrates, marks it for ubiquitination, and is both necessary and sufficient for its ubiquitination in an *in vitro* assay. Ozz binding to MyHC_emb_ differs from that of other E3 ligases in that it targets the tail portion of assembled sarcomeric myosin rather than the head portion of soluble myosin, and we suggest that these properties are fundamental to its role in muscle development as opposed to muscle atrophy.

MyHC_emb_ is the majority myosin isoform in embryonic and neonatal muscle fibers and its expression declines after birth to become undetectable around 3 weeks postnatal [20]. This postnatal decline is a robust process, not affected in null mutants of other myosin isoforms [20] or in animals where development of adult isoforms is retarded by undernutrition [36]. However, we found that the decline is slowed in a model system of differentiating *ozz^−/−^* primary myoblasts and in postnatal *ozz^−/−^* muscles.

During myofibrillogenesis, the developmental exchange of MyHC isoforms requires a myosin molecule to be released from its complex insertion into a sarcomeric thick filament, in order to be replaced by a subsequently expressed isoform. Davis, in a model of this process [2], concluded that core myosin molecules within a myofilament are essentially inaccessible to exchange by mass action and that a “facilitated exchange” process must exist in order to account for the rapid and complete change of isoforms observed *in vivo*. Without an additional regulatory process, exchange at equilibrium would be limited to the exchange of subunits away from the center of the filament.

**Figure 8:**
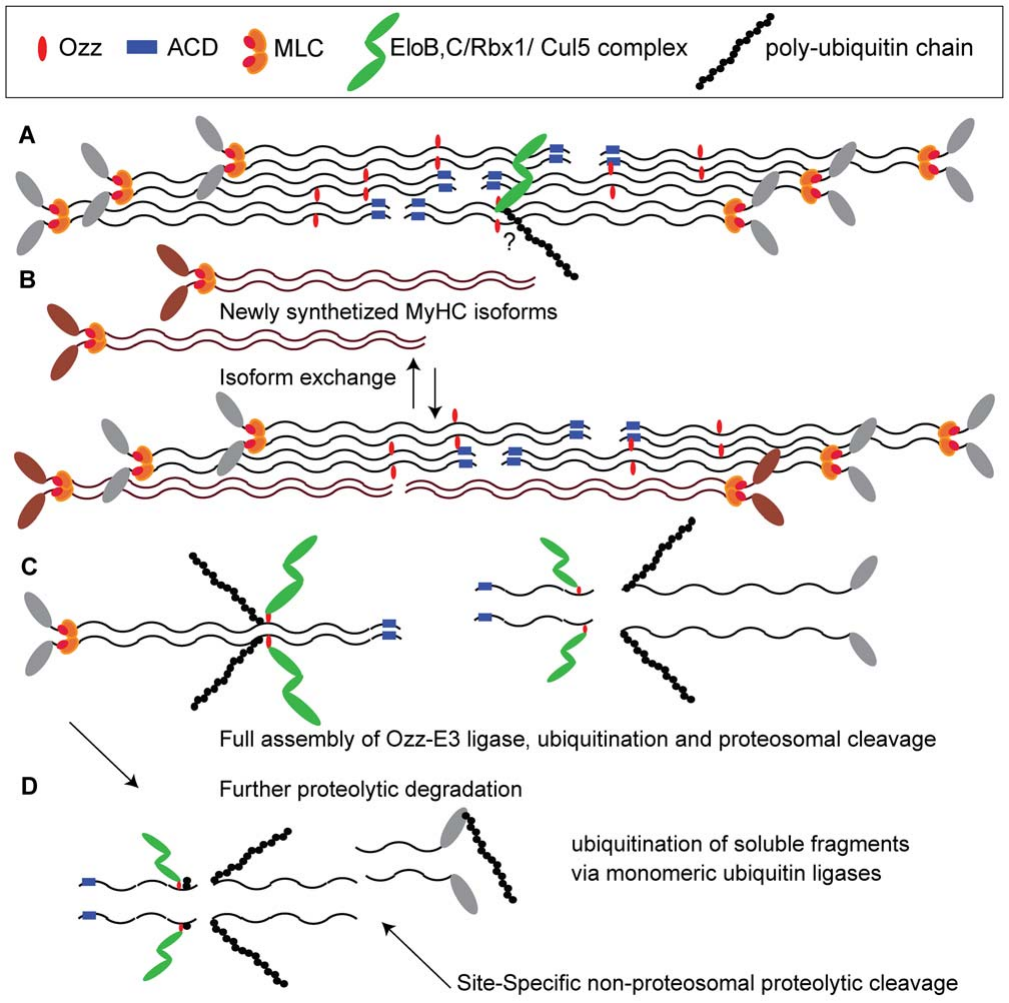
Model of the Sequence of Events Involved in Ozz-Mediated MyHC_emb_ Degradation from Sarcomeric Thick Filaments. (A) Ozz is bound to the rod portion of sarcomeric MyHC_emb_ in the vicinity of residue 1535, a possible region for lateral binding with the ACD residues on adjacent myosin rods [43]. Exchange between sarcomeric MyHC_emb_ and newly synthesized neonatal MyHC may be initiated by full Ozz-E3 ligase assembly and initiation of ubiquitination of sarcomeric MyHC_emb_, as demonstrated by co-immunoprecipitation of Ozz-Elo C from sarcomeric myosin, and the presence of ubiquitinated MyHC_emb_ in sarcomeric myosin preparations. (B) Isoform exchange, releasing soluble MyHC_emb_ by now having a full Ozz-E3 ligase assembled (C), leading to its prompt ubiquitination and proteolytic cleavage, resulting in the absence of Ozzylated MyHC_emb_ in the soluble myosin pool. (D) Further proteolytic degradation, involving specific proteases and ubiquitination of soluble fragments mediated by monomeric ubiquitin ligases, e.g. MuRF family members.

We found that Ozz is associated with sarcomeric but not soluble MyHC_emb_ from the earliest stages of muscle formation. The fact that Ozz and its direct partner Elo C could be co-immunoprecipitated from sarcomeric but not soluble myosin extracts of E16.5 embryonic muscle indicates that at this age a proportion of Ozz molecules bound to MyHC_emb_ is assembled into the E3 ligase complex. We also demonstrated that formation of such a complex is sufficient for *in vitro* ubiquitination of sarcofilamentous MyHC_emb_. From these findings, we can infer that the orderly removal of assembled MyHC_emb_ is achieved by tagging it with the Ozz-E3 ubiquitin ligase (Fig. 8). This refined mechanism, exchanging single molecules within a macromolecular assembly, would enable isoform exchange without any necessity for demolition and reconstruction within the cell, and is expected to represent a principle, which may be exploited by other subcellular systems. Moreover, our findings complement recent work on myosin assembly, which requires the coordinated action of chaperones and ubiquitin ligases [28], [37] to construct multimeric myosin ready for insertion into sarcomeric thick filaments.

Myosin assembly into thick filaments depends on a 29 amino acid assembly competence domain (ACD) near the C-terminal end of the myosin rod domain [26]. We found that Ozz recognizes the rod portion of MyHC_emb_, which forms the core of sarcomeric thick filaments where it is not easily accessed for binding or exchange by soluble cytoplasmic molecules. This is in contrast to the other known myosin E3 ligases, which target the head region of myosin [9], the portion of myosin exposed for interaction with actin and ATP. Furthermore, they appear able to access only sarcoplasmic, not sarcomeric myosin [9], [38], and their E3 ligase activity does not require further activation once they have recognized their substrate.

We hypothesize that during muscle fiber differentiation sarcomeric MyHC_emb_ becomes ubiquitinated by the Ozz-E3, and dissociates from the rest of the sarcomere. We showed that Ozz and Elo C are present together with sarcomeric myosin from the earliest stages of myofibrillogenesis, but we saw no obvious increase in Ozz/MyHC_emb_ ratio in association with the onset of peak periods of MyHC_emb_ degradation during embryogenesis (e.g. Fig. 3C *vs*. Fig. 3A, 3B). On the basis of these results, we conclude that Ozz-E3 ligase promotes the ubiquitination and the degradation of sarcomeric MyHC_emb_ (Fig. 8).

However, we cannot exclude that this process may occur in two phases. This alternative model implies that, in contrast with the action of one-chain ligases, Ozz does not immediately initiate ubiquitination and proteolytic degradation but may have a chaperone-like function in a preassembled form and only subsequently may gather the rest of the complex and become an active ligase. This two-step mode of action of the Ozz-E3 may explain the timely and regulated replacement/exchange of myofilamentous myosin during muscle differentiation and regeneration. This model of regulated assembly and disassembly of MyHC could be envisaged also for the adult muscle, where Ozz is expressed at basal levels [12]. In the adult muscle the Ozz-E3 activity would ensure the maintenance of myofiber integrity and the regulated exchange of isoforms under stress fiber conditions.

Modulation of cell structure by disaggregation and reassembly of cytoskeletal subunits is common to all cells, as is ubiquitination of many of the signaling proteins that control this process. The ubiquitin system also regulates signaling proteins controlling cell metabolism, cell cycle, and ion channel turnover, and is involved in chaperone-mediated myosin assembly [27]–[30]. It has a prominent role in recognizing misplaced or damaged proteins and promoting their degradation, and has a particular association with muscle atrophy. However, Ozz is upregulated during development, growth and regeneration, and downregulated in atrophy, and represents to our knowledge the first E3 ligase to be described as having a role in targeting myosin in its fully assembled sarcomeric structure during muscle remodeling. The Ozz model may be a paradigm for other developmental systems requiring protein isoform exchange within complex cytoskeletal structures.

## Materials and Methods

### Yeast Two-Hybrid Screening

Screening of an E14.5 mouse cDNA library (gift of P. McKinnon) for putative Ozz-binding partners was performed as previously described [12]. Three cDNA clones encoding the tail portion of MyHC_emb_ were isolated. To confirm the absence of interaction with the head and neck portion of MyHC_emb_ the corresponding prey construct (1–1047 aa) cDNA was amplified using a 10 µl reverse transcription reaction with primers 5′-GAGGGTGGGTCGACCATGAGTAGCGACACCGAGATGG-3′ and 5′-TACTTAGCGGCCGCTCATTGTTCGAGGGAGCTCTCCAG-3′. The RT-PCR product was cut with *Sal*1 and *Not*1 and inserted into the prey vector pEXP-AD502 in frame with the GAL4 activation domain. The deletion mutants of the MyHC_emb_ tail region were amplified using appropriate primers (see below). These PCR fragments were digested with *Sal*1 and *Not*1 and subcloned into the prey vector pEXP-AD502 in frame with the GAL4 activation domain. For yeast two hybrid assays, the bait and prey constructs were co-transformed into yeast strain Mav203. Two reporter genes (HIS3 and LacZ) were employed to study the protein (Invitrogen).

### Primers

MyHC_emb_ (1042–1535 aa): Forward GAGGGTGGGTCGACCAAGAAGCTGCGTGTGGA

CCTA; Reverse TACTTAGCGGCCGCTCACTCCATCTGTTTTCTGGATTT.

MyHC_emb_ (1536–1941 aa): Forward AGGCTTGTCGACCCTGGAGAAGGCTGACATCC

AGA; Reverse CGCGTGCGGCCGCTCACTCCTCGCTTTCATGGACCAC.

MyHC_emb_ (1738–1941 aa): Forward AGGCTTGTCGACCCTCCAGAGTGAGGTGGAGG

AT; Reverse CGCGTGCGGCCGCTCACTCCTCGCTTTCATGGACCAC.

MyHC_emb_ (1536–1871 aa): Forward AGGCTTGTCGACCCTGGAGAAGGCTGACATCCA

GA; Reverse CGCGTGCGGCCGCTCATTTATCCACCAGATCCTGCAA.

MyHC_emb_ (1872–1941 aa): Forward GGCTTGTCGACCCTCCAAGTGAAAGTCAAGTCC;

Reverse CGCGTGCGGCCGCTCACTCCTCGCTTTCATGGACCAC.

MyHC_emb_ (1901–1941 aa): Forward GCTTGTCGACCCAGCATGAGCTAGAGGAGGCC

Reverse CGCGTGCGGCCGCTCACTCCTCGCTTTCATGGACCAC.

### Surgical Procedures

All animal experiments were performed according to animal protocols approved by our Institutional Animal Care and Use Committee and National Institutes of Health guidelines. C57Bl/6 wild-type and *ozz*-null mice [12] were anaesthetized with isofluorane, and following surgery received ibuprofen, 7.5 mg/kg daily, for pain relief. Muscles of the lower hind limbs were denervated by sectioning the sciatic nerve in the lower thigh, just above the common peroneal–tibial nerve junction. Alternatively, they were tenotomised by sectioning the Achille's tendon. Muscle degeneration–regeneration was induced by injecting 10 mM cardiotoxin into the *gastrocnemius* (100 µl) or *tibialis anterior* (30 µl) muscles of 1–3 month-old mice. All experimental muscles were in the right limbs, with the left used as controls. Mice were sacrificed by CO_2_ inhalation before dissection.

### Isolation of Embryonic Muscles

Dated pregnant mice were sacrificed by CO_2_ inhalation, and the uterus quickly removed and placed on ice. Individual embryos were pinned on a Sylgard dish and dissected under ice-cold saline solution. The viscera, skin and spinal cord were removed and the embryo decapitated, leaving muscles of all four limbs, ribcage and back, with their associated cartilage. Tissues were snap frozen in liquid nitrogen.

### Cell Cultures

Myoblast cultures were established as described previously [12], [39], [40].

### Antibodies and Reagents

Rabbit anti-Ozz antibody was prepared as described [12]. The antibody was diluted 1:500 for Western blotting and 1∶10 for immunofluorescence. Mouse monoclonal antibodies anti-MyHC_emb_ (F1.652) 1∶400, anti-MLC (T14) 1∶500, and anti-pan myosin (MF20) 1∶500 were purchased from the Developmental Studies Hybridoma Bank. Anti-MyHC_emb_ (2B6) 1∶500, was a gift from Dr. N. Rubinstein. Other commercial antibodies included mouse anti-GST 1∶500 (UPSTATE), mouse anti-ubiquitin 1∶500 (Zymed), anti-Elo C 1∶300 (BD Biosciences), anti-GAPDH 1∶5000 (Millipore), rabbit anti-Elo B 1∶300 (Santa Cruz), anti-Rbx-1 1∶500 (Thermo Scientific), anti-Cul5 1∶200 (Santa Cruz), anti-pan actin 1∶5000 (Cell Signaling), normal mouse IgG (Santa Cruz), anti-Hsp 70 1∶4000 (Novus Biologicals), Cy3-conjugated anti mouse IgG and Cy3-conjugated anti-rabbit 1∶500 (Jackson ImmunoResearch), Alexa Fluor 488-conjugated anti-mouse 1∶500 (Invitrogen), FITC-Phalloidin 1∶500 (SIGMA-ALDRICH) and MG-132 (Enzo Life Sciences).

### Co-immunoprecipitation

Ozz-MyHC_emb_ complexes were detected in crude lysates of myoblast by immunoprecipitation with anti-MyHC_emb_ antibody, followed by Western blotting of the immunoprecipitates with the anti-Ozz antibody. Cultured myoblasts were lysed (lysis buffer: 50 mM HEPES (pH 7.5), 150 mM NaCl, 1% Igepal CA-630, 0.5% deoxycholate, 0.1% SDS, 5 mM EDTA, 1 mM EGTA, 1 mM DTT, 1 mM PMSF, protease inhibitors, phosphatase inhibitor), incubated at 4°C for 30 min, and cellular debris were pelleted by 20 min centrifugation at 12000 x g. The lysate was pre-cleared by 1 hr incubation at room temperature with 30 µl of Gamma Bind Plus Sepharose beads (Amersham Biosciences) and then spun at 1500×g. Anti-MyHC_emb_ (2B6) or anti-Ozz antibody was added to the supernatant and incubated for 1 hr at room temperature, followed by immunoprecipitation with Gamma Bind Plus Sepharose beads and overnight incubation at 4°C. The beads were washed 3x with lysis buffer, and 1x with lysis buffer without detergents, and the bound proteins detached and run on SDS-polyacrylamide gels under denaturing conditions.

### Fractionation of Muscle Tissues and Co-immunoprecipitation

Muscles from E14.5, E16.5, and E18.5 embryos were homogenized for 60s in a Dounce homogenizer in 4 volumes of lysis buffer [28] (Tris-HCl, 20 mM, pH 8.0; NaCl, 200 mM; MgCl_2_, 5 mM; DTT, 5 mM) and centrifuged at 16000×g for 5 min. The supernatant was then spun at 100,000×g for 2.5 hr at 4°C. The resulting supernatant was kept as the S fraction (cytoplasmic) and the pellet as the P fraction (sarcomeric). The P fraction was resuspended in 600 µl of lysis buffer.

S and P fractions prepared from E16.5 embryos were immunoprecipitated with anti-Elo C or anti-MyHC_emb_ using Protein G Dynabeads (Invitrogen), as described above.

### Gel filtration Columns of S and P Fractions

S and P fractions were separated on a Superose 6 gel filtration column (GE Healthcare). Aliquots from the gel filtration column were then denatured and run on SDS-polyacrylamide gels, followed by Western blotting with the appropriate antibodies. For calculation of the molecular weight the column was calibrated with the following proteins: thyroglobulin, 669 kDa; apoferritin, 443 kDa; β-amylase, 200 kDa; carbonic anhydrase, 29 kDa (SIGMA). At least 54 aliquots were analyzed, giving a size resolution from ≈2000 kDa to <10 kDa.

### In vitro Ubiquitination of GST-MyHC_emb_ Fragment

Four µg of a bacterially expressed GST-MyHC_emb_ fragment (1041–1941 aa) was incubated with 150 ng of purified recombinant E1 (Calbiochem), 200 ng UbcH5b (a gift of Dr. B. Schulman), 1.0 µg Ozz-E3 ubiquitin ligase and 7.5 µg of ubiquitin (Calbiochem) or K48R ubiquitin (Calbiochem) in a final volume of 30 µl of ubiquitination buffer (0.05M Tris-HCl, pH 7.6; 0.01M MgCl_2_, 0.004M ATP) at 30°C for 60 min.

To analyze the ubiquitinated products, 4.0 µg of GST-MyHC_emb_ fragment (used as control) or the ubiquination reaction mixtures were diluted in 500 µl of RIPA buffer (50 mM Tris HCl (pH 7.5), 150 mM NaCl, 1% NP–40, 0.1% deoxycholate, 0.1% SDS, 1 mM EDTA, protease inhibitors and phosphatase inhibitors), immunoprecipitated with anti-MyHC_emb_ (2B6), resolved on a 7.5% SDS-gels, and immunoblotted with either anti-MyHC_emb_, anti-ubiquitin, or anti-GST antibodies (Zymed).

### Purification and *In Vitro* Ubiquitination of Native MyHC

Sarcomeric MyHC from P1 muscle was purified as described [33], [34]. Purified MyHC (3 µg) was subjected to *in vitro* ubiquitination following the conditions described above. The reaction mixtures were resolved on a 7.5% SDS-polyacrylamide gel and immunoblotted with anti-ubiquitin antibody (Zymed).

### Thin-Thick Filaments

Muscle sarcomeric thin-thick filaments were purified according to the procedure of Trinick et al. [31].

### Western Blotting

Protein concentrations were determined as OD 595, using BSA as standard. 25 µg of soluble protein or 6.25 µg of insoluble protein were electrophoresed (100 V, 60 min) on SDS- gradient gels (NuPAGE 4–12% Bis-Tris Gel, Invitrogen), and wet-blotted overnight at 30 mA. Membranes were probed with specific antibodies at the dilutions listed above, followed by HRP conjugated goat anti-rabbit or anti-mouse IgG (Jackson ImmunoResearch Laboratories). Signals were detected with a West Femto maximum sensitivity substrate kit (Thermo Scientific) on a molecular imager (ChemiDoc XRS, BioRad). Each of the immunoblots included in the Figures was representative of results obtained in at least three independent experiments.

Immunoblots were photographed in a BioRad Chemidoc XRS Molecular Imager, and, where appropriate, band densities measured using BioRad Quantity One software. Montages were assembled using Adobe Illustrator, and then converted to TIFF files.

### Immunofluorescence and Imaging

Immunofluorescence analyses were performed on cultured myotubes (day 4) treated and not treated with proteasome inhibitor. MG-132 was added to the culture medium at a final concentration of 5 µM for 6 hr at 37°C. Myotubes were fixed in 3% PFA and immunostained with anti-Ozz and anti MyHC_emb_ antibodies. Cy3 anti-rabbit IgG (Jackson Laboratories) and Alexa Fluor 488 anti-mouse IgG (Invitrogen) were used as secondary antibodies. Analysis of the fluorescence intensity of MyHC_emb_ was performed on cultured myotubes (day 4). In this experiment Cy3 anti-mouse IgG (Jackson Laboratories) was used as secondary antibody.

Immunofluorescence analyses of MyHC_emb_ expression in muscle tissue were performed on hind limb muscles from wild-type and *ozz^−/−^* P7.5 pups. Muscles were embedded in OTC freezing solution and sectioned sequentially from the distal tendinous insertion towards the mid belly region. Cross sections were labeled with anti-MyHC_emb_ and FITC-Phalloidin (SIGMA) followed by incubation with Cy3-conjugated secondary antibody (Jackson ImmunoResearch) prior to confocal microscopy imaging.

Images were acquired on a Nikon C1si confocal microscope, with a Plan Apo 40X, NA 1.3 and/or Plan Apo 60X, NA 1.45 objective (Melville, NY).

### Calculation of Co-localization Coefficients

Computational analyses of confocal images were performed with the NES-Elements AR 3.1 (Melville, NY). Pearson's correlation coefficient (Rr), Manders overlap (R), and Manders overlap coefficients *k1* and *k2* were employed to evaluate the extent of co-localization of the two fluorescent dyes. Pearson's correlation coefficient (Rr) is one of the standard measures in pattern recognition. It is used for describing the correlation of the intensity distributions between channels. It takes into consideration only similarity between shapes while ignoring the intensities of signals. Its values range is between -1.0 and 1.0, where 1.0 indicates no overlap and 1.0 is a complete co-localization. Manders overlap coefficient is a generally accepted measure of co-localization. It indicates an overlap of the signals and thus represents the true degree of co-localization. Values of the R are defined from 0 to 1.0. If an image has an overlap coefficient equal to 0.5, it implies that 50% of both its components overlap with the other part of the image. A value of zero means that there are no any overlapping objects Overlap coefficients *k1* and k*2* split the value of co-localization into two separate parameters. *k1* and k*2* coefficients depend on the sum of the products of the intensities of two channels. Thus, they are sensitive to the differences in the intensity of two signals and should be used accordingly [41], [42].

### Statistical Analysis

Data were expressed as mean ± SD and evaluated using Student's t-test for comparison with wild-type samples. Mean differences were considered statistically significant when P values were less than 0.05 (*).

## Supporting Information

Figure S1 (A) Yeast 2-Hybrid Screen Demonstrating that Ozz Interacts with the Tail Portion of MyHC_emb_ (1047-1941 aa) but not the Head and Neck (1-1041 aa).
(8.67 MB TIF)

Figure S2 (A) Immunofluorescence analyses of the expression of MyHC_emb_ in ozz knock-out and wild-type differentiated myoblast (day 4). (B) Quantification of intensity of the expression of MyHC_emb_ in the differentiated myoblast.
(5.45 MB TIF)
