# Supplementary figures and images for: Ozz-E3 Ubiquitin Ligase Targets Sarcomeric Embryonic Myosin Heavy Chain during Muscle Development
Source: PLoS One. 2010 Mar 24;5(3):e9866. doi: 10.1371/journal.pone.0009866 (PMC2844429; doi:10.1371/journal.pone.0009866)

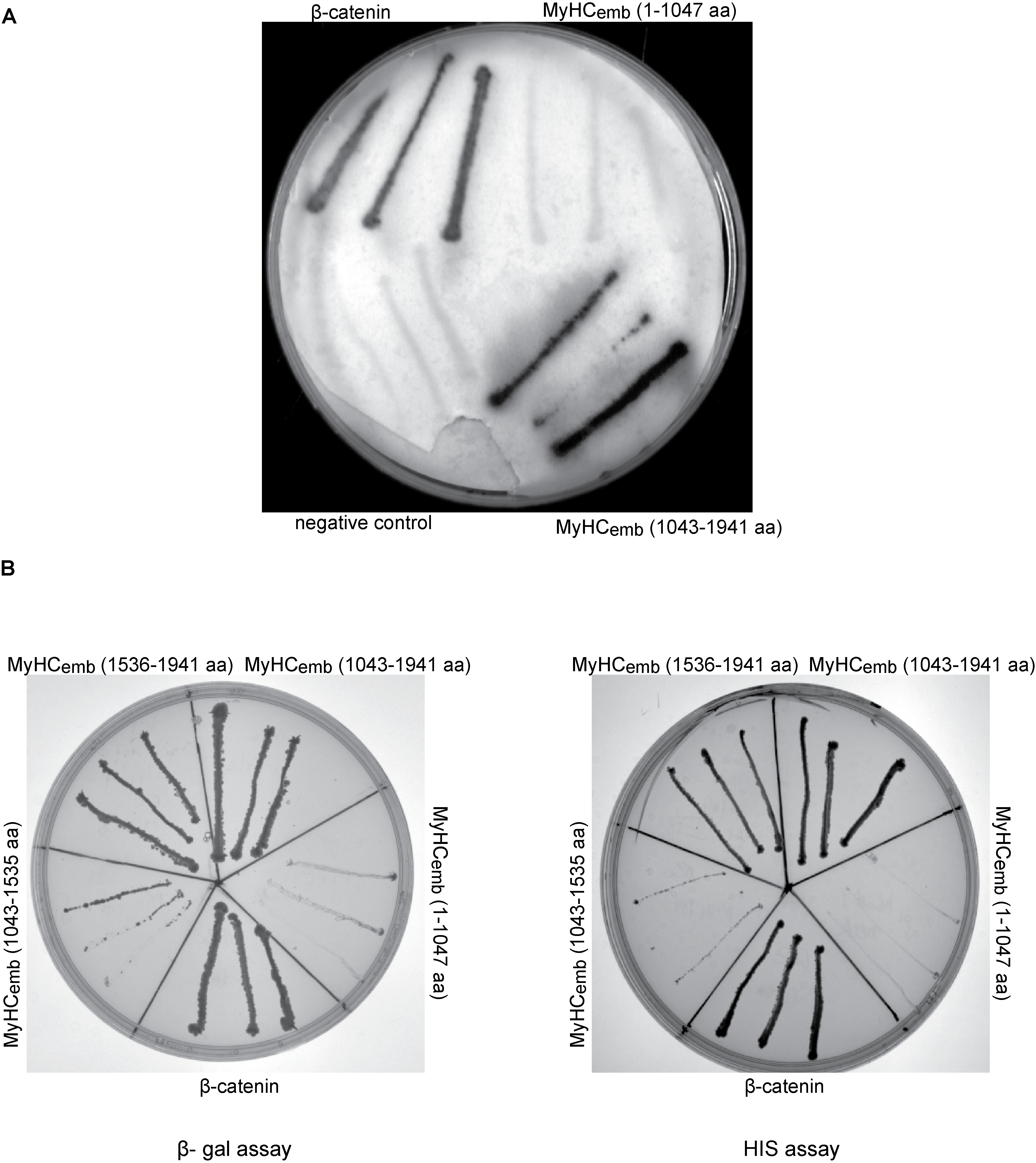

Supplement: Figure S1 — (A) Yeast 2-Hybrid Screen Demonstrating that Ozz Interacts with the Tail Portion of MyHCemb (1047-1941 aa) but not the Head and Neck (1-1041 aa). (8.67 MB TIF) [file pone.0009866.s001.tif]

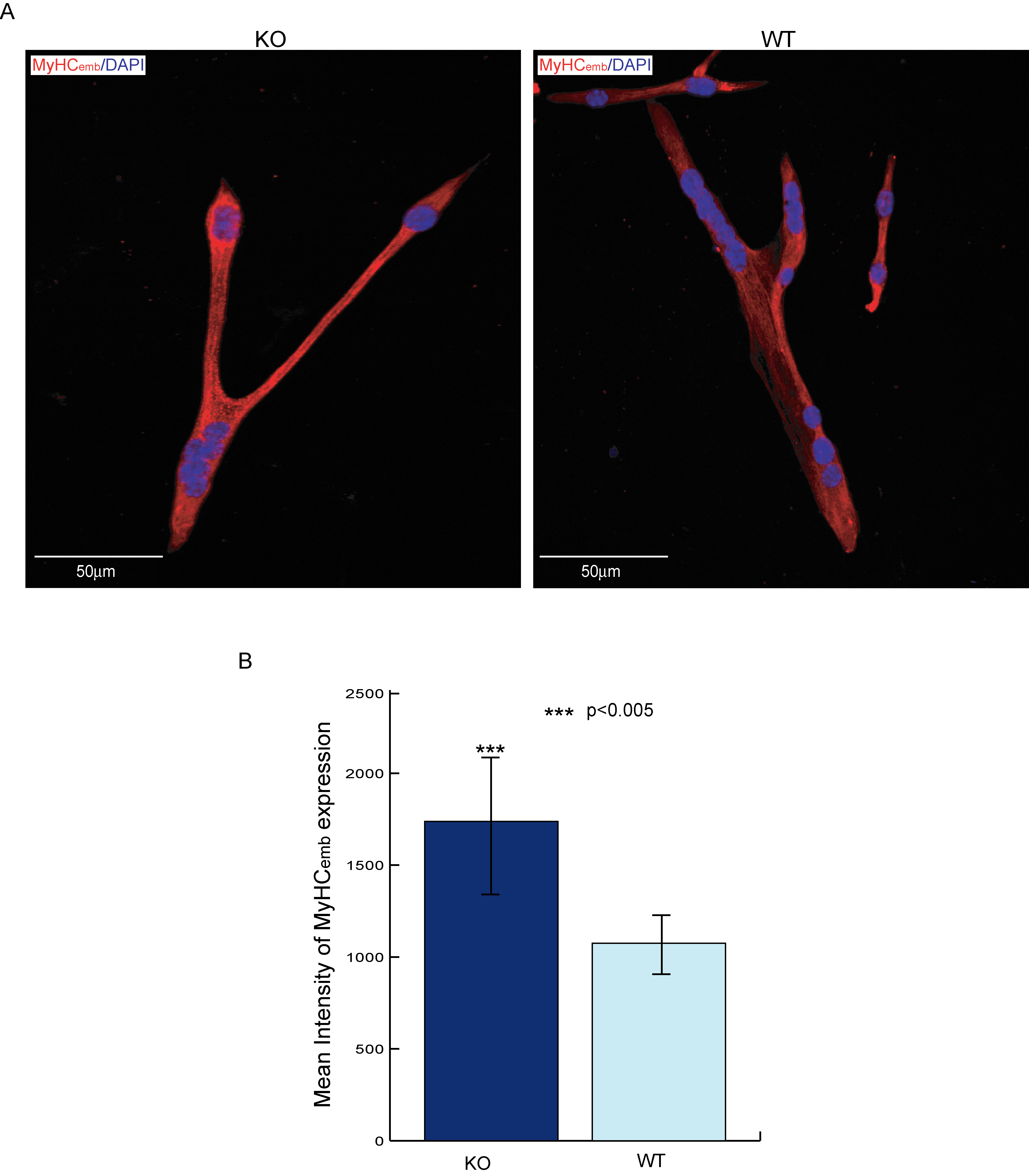

Supplement: Figure S2 — (A) Immunofluorescence analyses of the expression of MyHCemb in ozz knock-out and wild-type differentiated myoblast (day 4). (B) Quantification of intensity of the expression of MyHCemb in the differentiated myoblast. (5.45 MB TIF) [file pone.0009866.s002.tif]
